# Statins and risk of type 2 diabetes: mechanism and clinical implications

**DOI:** 10.3389/fendo.2023.1239335

**Published:** 2023-09-19

**Authors:** Markku Laakso, Lilian Fernandes Silva

**Affiliations:** ^1^ Institute of Clinical Medicine, Internal Medicine, University of Eastern Finland, Kuopio, Finland; ^2^ Kuopio University Hospital, Kuopio, Finland

**Keywords:** statin, type 2 diabetes, glucose, insulin secretion, insulin resistance

## Abstract

Statins are widely used to prevent cardiovascular disease events. Cardiovascular diseases and type 2 diabetes are tightly connected since type 2 diabetes is a major risk factor for cardiovascular diseases. Additionally, cardiovascular diseases often precede the development of type 2 diabetes. These two diseases have common genetic and environmental antecedents. Statins are effective in the lowering of cardiovascular disease events. However, they have also important side effects, including an increased risk of type 2 diabetes. The first study reporting an association of statin treatment with the risk of type 2 diabetes was the WOSCOPS trial (West of Scotland Coronary Prevention Study) in 2001. Other primary and secondary cardiovascular disease prevention studies as well as population-based studies have confirmed original findings. The purpose of our review is to examine and summarize the most important findings of these studies as well as to describe the mechanisms how statins increase the risk of type 2 diabetes.

## 1 Introduction

Several risk factors are shared between type 2 diabetes (T2D) and cardiovascular disease (CVD), obesity, dyslipidaemia, insulin resistance, and hyperglycaemia. T2D is a major risk factor for CVD, and CVD often precedes the development of T2D. Thus, these diseases have common genetic and environmental antecedents (1).

**Figure 1:**
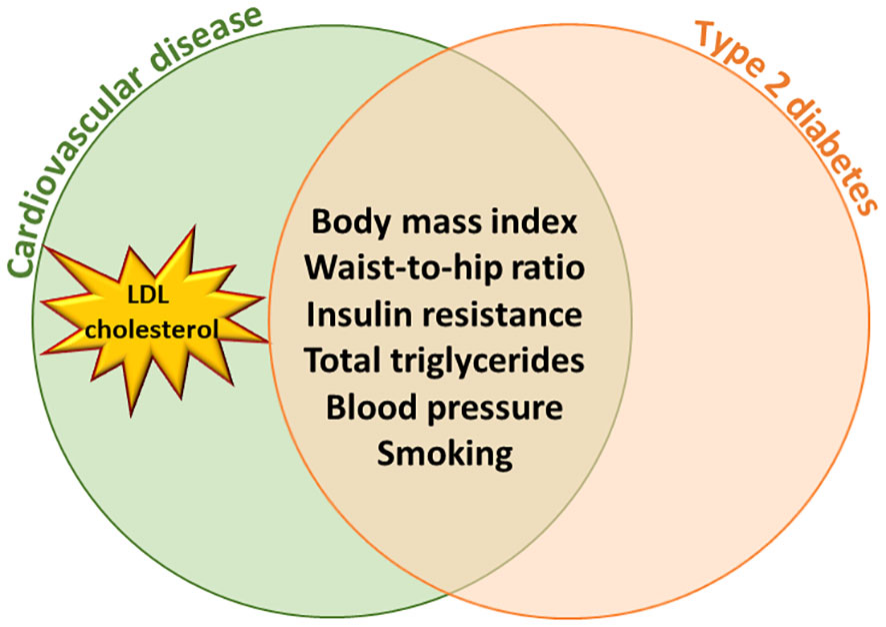
Mendelian Randomization studies have shown that body mass index, waist-to- hip ratio, insulin resistance, total triglycerides, blood pressure and smoking are causally associated with the risk of type 2 diabetes and cardiovascular diseases. High LDL cholesterol concentration is causally associated with cardiovascular diseases but not with type 2 diabetes.

Mendelian randomization (MR) approach uses a genetic instrument to infer causal associations between an exposure and an outcome (2). These studies have demonstrated that body mass index (BMI), waist-to-hip ratio (3), elevated blood pressure (4), smoking (5), and total triglycerides (6) are causally associated with the risk of both T2D and CVD. MR studies also indicate that T2D is causally associated with coronary artery disease independent of other risk factors (7, 8). However, a major difference is in low-density cholesterol concentrations (LDL-C) since high concentrations of LDL-C are causally associated with CVD (9) but not with the risk of T2D (**Figure 1**).

Statins were introduced for the first time in 1987 when lovastatin was approved in the United States. Simvastatin and pravastatin were approved in 1991, fluvastatin in 1994, atorvastatin in 1997, rosuvastatin in 2003 and pitavastatin in 2009. Several trials reported that statins are effective in the lowering of LDL-C (10, 11), and therefore statins are currently the most widely prescribed drugs in the primary and secondary prevention of CVD events (12). The maximum dose of rosuvastatin, atorvastatin and simvastatin lower LCL-C by 50-60%, lovastatin by 50%, and pravastatin and lovastatin by 30-40%. Statins inhibit 3-hydroxymethyl glutaryl coenzyme A (HMG-CoA) reductase, an intracellular enzyme catalysing the conversion of HMG-CoA to mevalonate during the rate-determined step of cholesterol metabolism (13, 14). However, statins have side effects, especially hyperglycaemia and the risk of T2D. Therefore, the United States Safety and Drug Administration released changes in statin safety label in 2012 that statins increase glycosylated haemoglobin A1c (HbA1c) and fasting glucose concentrations (15).

The first studies suggesting that statins increase the risk of T2D came from statin trials (16). The benefit of trials is that they include large number of participants. However, trial data have important limitations. The hypothesis in statin trials was that statins reduce the risk of CVD by lowering LDL-C concentrations. The primary endpoints in these trials were incident CVD events, and not conversion to T2D according to the criteria of the American Diabetes Association (17). Therefore, it is impossible to make reliable conclusions from these trials with respect to incident T2D. Participants needed to have elevated LDL-C concentrations and a high risk of CVD, and therefore they differed from the general population. Furthermore, most meta-analyses combined primary and secondary prevention populations which is problematic because secondary prevention populations include survivors of CVD. Additionally, the diagnosis of T2D was often evaluated *post hoc* and based on self-reported T2D or physician diagnosed T2D without internationally accepted criteria for diabetes. In none of these trials the diagnosis of T2D was based on the measurements of fasting and 2-hour glucose concentrations and HbA1c according to the American Diabetes Association criteria (17) resulting in underestimation of cases of T2D (18).

## 2 The first evidence that statins increase the risk of T2D

The first study showing that statins were associated with the risk of T2D was the WOSCOPS trial (West of Scotland Coronary Prevention Study) (19). A total of 5 974 participants did not have diabetes at baseline and 139 of them developed T2D during the study. Pravastatin therapy resulted in a 30% reduction of the cases of T2D in a *post hoc* analysis of this study. The diagnosis of T2D was based only on fasting glucose measurements. The PROSPER trial (pravastatin in elderly individuals at risk of vascular disease) included 5 804 participants and reported a 19% decrease in the risk of coronary artery disease, but no significant increases or decreases in incident T2D (20). The PROVE-IT TIMI 22 sub-study reported for the first time increases in glucose and HbA1c concentrations and incident T2D for atorvastatin (21), and the Phase Z of the A to Z trial for simvastatin (22).

The JUPITER trial (Justification for the Use of Statins in Prevention: Intervention Trial Evaluating Rosuvastatin) randomly assigned 17 802 men and women to rosuvastatin 20 mg/daily. The participants had LDL-C < 3.4 mmol/l and high-sensitivity C-reactive protein levels of 2.0 mg/l or higher (23). Myocardial infarction, stroke, arterial revascularization, hospitalization for unstable angina, or death from CVD formed the primary endpoint of this trial. Rosuvastatin significantly lowered CVD events by 44%, and increased incident physician-reported T2D by 26% compared to the placebo group.

Sattar et al. (24) published the first large meta-analysis of statin-induced incident T2D. This meta-analysis included 91140 participants from13 trials. Statin therapy increased the risk of incident T2D by a 9%. Preiss et al. (25) reported in another meta-analysis including 32 752 participants from five statin trials that there was a significant increase in fasting glucose levels and that the risk of T2D increased by 12%. In this study high intensity statin trials (atorvastatin, rosuvastatin, simvastatin) were more often associated with incident T2D than moderately intensity statins (pravastatin, pitavastatin). Carter et al. published similar results in a population-based study (26). Thakker et al. (27) reported the results of a meta-analysis including 141 863 participants without diabetes from 29 trials and found that 12% of the participants developed T2D.

Early findings from statin trials showed that that pre-existing risk factors for T2D, including age, obesity, total triglycerides, and blood pressure, increased the development of statin-mediated hyperglycaemia. Data from the Treating to New Targets (n=7 595) and IDEAL (n=7 461) trials reported that increased fasting glucose (≥5.6 mmol/l) and the components of the metabolic syndrome increased the risk of T2D (28–30). Of the patients having high statin dose (atorvastatin, simvastatin) and 2-4 risk factors for T2D, 14,3% developed T2D whereas from the patients on a lower-dose statin dose 11,9% developed T2D. Thus, the dose-related effects of statins may depend also on the general risk factors for T2D (28). Additionally, in meta-analyses of large clinical trials other drugs used for the prevention of CVD events, niacin, thiazide diuretics and beta-blockers increased the risk of T2D from 9% to 43% (31).

In summary, the first statin trials and meta-analyses showed a decrease in the risk of T2D for pravastatin by 0-30%, and an increase in the risk of T2D by 12% for rosuvastatin, atorvastatin and simvastatin. The risk of T2D was somewhat greater for high-intensity statins (rosuvastatin, atorvastatin, simvastatin) compared to low- and medium-intensity statins. Importantly, an increased risk of incident T2D by statins was comparable with the risk of T2D of other drugs (beta-blockers and thiazide diuretics) used for the prevention of CVD.

## 3 Effects of statin therapy on glucose control in patients with previously diagnosed T2D

Zhou et al. (32) investigated the effects of statin treatment on glucose control in a meta-analysis including 3 232 participants having previously diagnosed T2D from 26 eligible studies. The investigators found that statin therapy did not have significant effects on glycaemic control (HbA1c, fasting glucose) in these patients. The results of Cui et al. (33) were different. They investigated HbA1c changes in 23 trials including 2 707 patients with previously diagnosed T2D and found a small but significant increase in HbA1c when statins were compared with placebo. High-intensity atorvastatin worsened glycaemic control whereas moderate-intensity pitavastatin improved it (33).

Erqou et al. (34) analysed the results from 9 696 participants (4 980 on statin, 4 716 controls) included in nine trials. The mean HbA1c of the participants randomized to statin treatment was modestly but significantly increased by 0.12% compared to the control group after an average follow-up of 3.6 years. Alvarez-Jimenez et al. (35) performed a meta-analysis of HbA1c concentrations based on 67 studies including over 25 000 patients with T2D. Statin increased HbA1c by 0.21%.

Mansi et al. (36) investigated the association of statin treatment initiation and diabetes progression in 83 022 statin users and non-users. The investigators found that statin users had a higher likelihood to start insulin treatment, develop significant hyperglycaemia, and a need to increase additional glucose-lowering medication. High-intensity LDL-C lowering medication was associated with an increased likelihood of diabetes progression among statin users compared to non-users. This study gave important information about the effects of statin therapy on glycaemic control among patients with diabetes.

In summary, statins worsened hyperglycaemia in patients with previously diagnosed T2D but increases in HbA1c were moderate. Statin users had an increased risk for T2D progression and insulin treatment initiation.

## 4 Statins and incident T2D in population-based studies

The limitation of previous statin trials is that the participants were selected among the individuals having a high risk of CVD. Therefore, the participants included in these studies do not represent the general population. Additionally, the diagnosis of T2D in previous studies was based often on self-reported diabetes or fasting glucose measurement that underestimate the incidence of T2D. The current diagnostic criteria for diabetes are the measurements of fasting and 2h glucose and HbA1c.

The first population studies indicated that the risk of statin-induced T2D was considerably higher than in statin trials. Carter et al. (26) conducted a population-based retrospective study including 471 250 patients treated with statin, aged 66 or older and without diabetes at baseline. A total of 38 470 patients were treated with pravastatin, 268 254 with atorvastatin, 11 923 with fluvastatin or lovastatin, 76 774 with rosuvastatin, and 75 829 with simvastatin. Compared with pravastatin, atorvastatin increased the risk of incident T2D by 22%, rosuvastatin by 18%, and simvastatin by 10%. Fluvastatin and lovastatin did not increase the risk of T2D compared to pravastatin. The strengths of this study were a large sample size, and a population-based design. The limitations were that this study did not have a control group of participants without statin treatment, and measurements of glucose or HbA1c. These findings are comparable to those of a study by Zaharan et al. (37) reporting that atorvastatin increased the risk of incident T2D by 25%, rosuvastatin by 42%, and simvastatin by 14%. In the Women’s Health Initiative study including 161 808 postmenopausal women without diabetes at baseline reported that statin therapy was associated with a 48% increase in the risk of self-reported diabetes (38).

Djousse L et al. (39) investigated the association of the effects of statin potency (low, medium, high) with incident T2D in 3 390 799 US Veterans. The authors reported that compared to no statin use low statin potency increased the risk of incident T2D by 21%, medium potency by 22%, and high statin potency by 34% (daily doses for simvastatin ≥80 mg, atorvastatin ≥40 mg, rosuvastatin ≥10 mg). This study shows that high doses of the most potent statins, simvastatin, atorvastatin, and rosuvastatin, increase further the risk of incident T2D.

We investigated the mechanisms associated with statin-induced T2D in the population-based Metabolic Syndrome in Men (METSIM) cohort (40, 41) including 8,749 non-diabetic participants, aged 45-73 years at baseline. During a 6-year follow-up we diagnosed incident T2D in 625 men. The criteria for diabetes were a need of glucose-lowering medication during the follow-up, elevated glucose concentrations in fasting or in an oral glucose tolerance test, or HbA1c ≥6.5% (48 mmol/mol). We found a 46% increased risk of T2D in the participants on statin treatment (n=2 142). Simvastatin and atorvastatin increased the risk of T2D in a dose-dependent manner. Statin treatment was additionally associated with decreased insulin sensitivity by 24% and deceased insulin secretion by 12% compared with the participants without statin treatment.

The prospective population-based Rotterdam Study included 8 567 participants without diabetes at baseline and a follow-up for 15 years (42). Statin treatment increased the risk of incident T2D by 38% after the adjustment for confounding factors. Diabetes was diagnosed by fasting glucose or a non-fasting glucose concentration ‗ 11.1 mmol/l. Subjects with impaired glucose homeostasis at baseline and obesity had the highest risk of T2D.

Crandall et al. (43) investigated incident T2D in the Diabetes Prevention Program Outcomes Study (n=3 234). This randomized clinical trial investigated the effects of different interventions to prevent T2D. The diagnosis of incident diabetes was based on an annual oral glucose tolerance test. At 10 years, the cumulative incidence of diabetes in the participants on statin treatment was 36% (20% in the placebo group, 33% in participants on metformin therapy, and 43% in the lifestyle group). This study was the first to investigate the statin-diabetes association within randomized clinical trial in the participants with high risk of diabetes. Statin use was associated with greater risk of T2D irrespective of the treatment group, with pooled 36% risk for incident T2D.

Engeda et al. (44) performed a meta-analysis of eight randomized controlled trials and 15 observational studies. The incidence of T2D in both randomized controlled trials and observational studies. Incident T2D was substantially larger in observational studies (55%) than in trials (11%).

In summary, several population-based studies have reported substantially higher number of incident cases of T2D among the individuals on statin treatment compared to previous CVD trials. This indicates that previous statin trials have largely underestimated the true incidence of T2D in patients on statin treatment.

## 5 Effects of genetic variants on statin function and the risk of T2D

Genetic variants in the three genes have effects on statin function, the solute carrier organic anion transporter family member 1B1 gene (*SLCO1B1*), 3-Hydroxy-3-Methylglutaryl-CoA Reductase (*HMGCR*), and Low-Density Lipoprotein Receptor (*LDLR*).

### 5.1 SLCO1B1

*SLCO1B1* gene encodes a liver-specific member of the organic anion transported family. The encoded protein organic-anion-transporting polypeptide 1B1 (OATP1B1) is highly expressed in the liver. The function of OATP1B1i s to transport statins and several other endogenous metabolites into the liver (45). Genetic variants in the *SLCO1B1* gene are associated with impaired transporter function (46). *SLCO1B1* rs4363657 variant is strongly associated with an increased risk of statin-induced myopathy (47).

We investigated the association of the effective C allele of *SLCO1B1* rs4149056 with the risk of T2D in the METSIM study (48). S*LCO1B1* rs4149056-C did not have significant association with the risk of T2D, glucose concentrations, insulin sensitivity, or insulin secretion. These findings suggest that OATP1B1-dependent transport of statins in the liver does not play a significant role in glucose metabolism. However, the participants on simvastatin treatment had higher fasting glucose, insulin, and proinsulin concentrations, lower LDL cholesterol concentration, and increased insulin resistance than the participants without statin treatment when we compared clinical and laboratory measurements between the carriers of the CC + CT genotype of *SLCO1B1* rs4149056.

We also investigated the effects of *SLCO1B1* rs4149056-C on metabolite concentrations in the participants on simvastatin treatment (n=1 373) and in age- and BMI-matched controls (n=1 368) without any statin medication. We found that concentrations of dicarboxylic acids were decreased in the participants with simvastatin. This may result in an increase of beta- and peroxisomal oxidation and increased turnover of cholesterol into bile acids. Consequently, steroidogenesis decreases attributable of limited availability of cholesterol for steroid synthesis (48).

In summary, *SLCO1B1* rs4149056-C did not have a significant association with the risk of T2D, elevated glucose concentration, insulin resistance, or impaired insulin secretion.

### 5.2 *HMGCR* gene

HMG-CoA reductase is the rate-limiting enzyme for cholesterol synthesis. It is regulated by a negative feedback mechanism mediated by sterols and non-sterol metabolites derived from mevalonate.

Statins inhibits the HMG-CoA reductase enzyme that suppresses the synthesis of mevalonate, cholesterol, and its downstream metabolites (48, 49). Several studies have clarified the significance of the *HMG-CoA* gene as a risk factor for T2D.

Swerdlow et al. (50) included in their analyses 223 463 individuals from 43 genetic studies. Each additional *HMGCR* rs17238484-G allele was associated with a mean 0.06 mmol/l lower LDL-C concentration and a 2% higher risk of T2D. Additionally, this variant was associated with higher body weight (0·30 kg), waist circumference (0·32 cm), plasma insulin concentration (1.62%), and plasma glucose concentration (0.23%). *HMGCR* rs12916-T allele was also associated with 6% higher risk of T2D, and quite similar effects on LDL-C concentration, weight, and waist circumference as *HMGCR* rs17238484-G allele. The authors concluded that the increased risk of T2D in individuals having statins treatment was at least partially explained by *HMGCR* inhibition, and weight gain.

Another recent GWAS meta-analysis included > 2 million participants of European and East Asian ancestry (51). Genetically mimicked effects of statins and ezetimibe were associated with higher risk of T2D, and BMI which was claimed to explain more than half of the effects of statins on the risk of T2D, in contrast to previously published results where the effects of BMI as a modified were modest (50). We did not observe increased weight gain in the participants on statin treatment who developed new T2D in our 6-year follow-up study of the METSIM cohort including 8 749 non-diabetic participants (40). Therefore, the role of weight gain and the effects of *HMGCR* variants as a causal factor for conversion to T2D is controversial (52, 53). It is important to remember that weight gain increases insulin resistance but does not have a direct effect on insulin secretion. Insulin secretion decreases when insulin secretion from pancreatic β-cells is not able to compensate for decreased insulin sensitivity. Therefore, in a previous study where insulin resistance increased insulin secretion increased correspondingly which is expected in studies having short follow-up (54).

In summary, *HMGCR* rs17238484-G allele increased the risk of T2D by 2%, body weight by 0·30 kg, waist circumference by 0·32 cm, plasma insulin concentration by 1.6%, and glucose concentration by 0.2%. These changes were small and therefore more studies are needed to confirm original results.

### 5.3 *LDLR* gene

Familial hypercholesterolemia is caused by homozygous or heterozygous pathogenic mutations in the *LDLR* gene (55, 56). Such mutations result in the expression of LDL receptors or transport of LDL-C into the cells (57). The association of familial hypercholesterolemia with a low risk of diabetes was first reported in 1997 (58). Fall et al. (59) reported a significant association between genetically increased circulating LDL-C concentrations and a decreased risk of T2D. However, the authors concluded that these associations could be caused by survival bias, pleiotropy, or unknown confounding factors and should be interpreted with caution. Lotta et al. (60) found an association between LDL-lowering genetic variants and T2D in a meta-analysis including 50 775 individuals with T2D and 60 801 individuals with coronary artery disease. However, the association of *LDLR* with the risk of T2D was not causal. Two other studies reported that patients with familial hypercholesterolemia have a decreased risk of diabetes (61, 62).

LDL-C is causally associated with coronary artery disease (63, 64). White et al. constructed a genetic instrument composed of 130 genetic variants for LDL-C (65). This genetic instrument was significantly associated with increased LDL-C concentrations as well as with an increased risk of coronary artery disease, odds ratio and its 95% confidence intervals for LDL-C were 1.68 (1.51-1.87). LDL-C was also associated with a decreased risk for diabetes, odds ratio and its 95% confidence limits were 0.79 (0.71-0.88). Therefore, clinical trials of lipid lowering agents should carefully monitor for glycemia and conversion to T2D (66).

In summary, accumulating evidence shows that elevated LDL-C concentration is inversely associated with decreased risk of T2D, but there is no firm evidence that this association is causal.

## 6 Mechanisms leading to incident diabetes with statin medication: human studies

We investigated the mechanisms underlying the risk of T2D associated with statin treatment in the METSIM cohort (40, 41) including 8 749 non-diabetic participants. New T2D was diagnosed in 625 men during a 6-year follow-up. We first validated our measurements of insulin sensitivity and insulin secretion in a separate sample of 287 non-diabetic individuals not belonging to the METSIM cohort. These individuals participated in an intravenous glucose tolerance test to evaluate insulin secretion and euglycemic-hyperinsulinemic clamp study to evaluate insulin sensitivity (67). We calculated 11 different indices for insulin secretion and 6 indices for insulin sensitivity. The ratio of insulin and glucose areas (from 0 to 30 min) under the curve (InsAUC_0-30_/GluAUC_0-30_) had the highest correlation (0.66) with the first phase insulin secretion and Matsuda index had the highest correlation (0.77) with the M value of the euglycemic hyperinsulinemic clamp. We found that the participants on statin treatment (n=2 142) had a 46% increased risk of T2D. Insulin sensitivity was decreased by 24% and insulin secretion by 12% in individuals on statin treatment. Our study shows that both insulin resistance and decreased insulin secretion were the mechanisms leading to the conversion to T2D (19).

Abbasi et al. (54) performed a clinical trial of atorvastatin 40 mg daily in 71 participants without CVD or T2D at baseline. The length of this study was 10 weeks. Atorvastatin increased insulin resistance by 8% and insulin secretion by 9% compared to the baseline measurements. In their study insulin secretion increased to compensate insulin resistance caused by atorvastatin during their trial. Our study lasted 6 years and therefore the pancreas was unable to compensate for increasing insulin resistance over a long time resulting in a decrease of insulin secretion (19).

In a meta-analysis of Baker et al. (68) pravastatin increased insulin sensitivity, atorvastatin and rosuvastatin did not affect insulin sensitivity and simvastatin decreased insulin sensitivity in participants without T2D. Alvarez-Jimenez et al. (35) investigated the effects of statin therapy on glycaemic control and insulin resistance based on a meta-analysis of 67 separate studies including > 25 000 participants. In participants with normal HbA1c (<6.5% or 48 mmol/l) rosuvastatin and atorvastatin induced significant increases in HbA1c in most of the studies. In participants having HbA1c > 6.5% only atorvastatin induced a significant increase in HbA1c. The same investigators analysed also changes in HOMA insulin resistance index. Rosuvastatin, simvastatin, and atorvastatin significantly increased insulin resistance in a subgroup having relatively low insulin resistance. The strength of this study is a large sample size, but HOMA-IR is not a reliable index for insulin resistance.

## 7 *In vitro* and *in vivo* studies on mechanisms leading to impaired insulin secretion and insulin resistance by statins

Both insulin resistance and impaired insulin secretion are hallmarks of T2D. Most of the genetic variants increasing the risk of T2D are regulating insulin secretion, and only few of insulin sensitivity (69). Decreased insulin secretion is the major contributor to statin-induced diabetes. Insulin production in the β-cells is controlled by several transcription factors playing crucial roles in the regulation of both the differentiation of β-cells into insulin-producing cells and β-cell function (70, 71). Therefore, understanding of the mechanisms resulting in disturbances in insulin secretion is important.

HMGCR increases modestly the risk of T2D (50). Takei et al. (72) deleted *Hmgcr* in a β-cell specific manner by using the Cre-loxP technique in mice. Mice lacking *Hmgcr* in β-cells exhibited low insulin concentrations and hyperglycemia attributable to decreases in both β-cell mass and insulin secretion. The β-cell mass reduction was mainly caused by impaired proliferation of β-cells. The investigators concluded that HMGCR plays critical roles not only in insulin secretion but also in the development of β-cells. These findings demonstrate the importance of the mevalonate pathway in the maintenance of β-cells and glucose homeostasis.

LDLR plays an important role in β-cell dysfunction. Statins reduce cholesterol synthesis via the HMG-CoA reductase pathway (70) resulting in increased cholesterol entry and accumulation in pancreatic β-cells, and impairment in β-cell function via glucose-induced Ca^2+^ signalling pathways (70, 73–76). In our study simvastatin decreased glucose-stimulated insulin secretion in MIN6 β-cells at normal glucose concentration by multiple mechanisms, including inhibitory effects on the acetylcholine and GPR40 pathways, whereas simvastatin-induced impairment in insulin secretion was substantially less in the GLP-1 receptor and GPR119 pathways (76). Interestingly, exenatide prevented statin related LDLR increase and improved insulin secretion in pancreatic β-cells (77). However, detailed mechanisms how statins inhibit cholesterol synthesis and lead to impaired L-type Ca^2+^ signalling function remains unclear (74).

The isoprenoids, products of mevalonate pathways are likely to be important for insulin secretion. Lovastatin decreased glucose-induced insulin secretion by 50% in rat islets, but co-incubation with mevalonate abolished this effect (78). Wang et al. (79) investigated the effects of simvastatin on glucose homeostasis in streptozotocin induced diabetic rats. They reported that statin therapy increased glucose concentrations over a period of 12 weeks. In another study the effects of simvastatin on insulin secretion were studied in mouse MIN6 cells and showed that high concentrations of simvastatin significantly reduced the synthesis and secretion of insulin compared to the control group (80). Similar results were also obtained in another simvastatin study in intact single-islet cultures (81).

**Figure 2:**
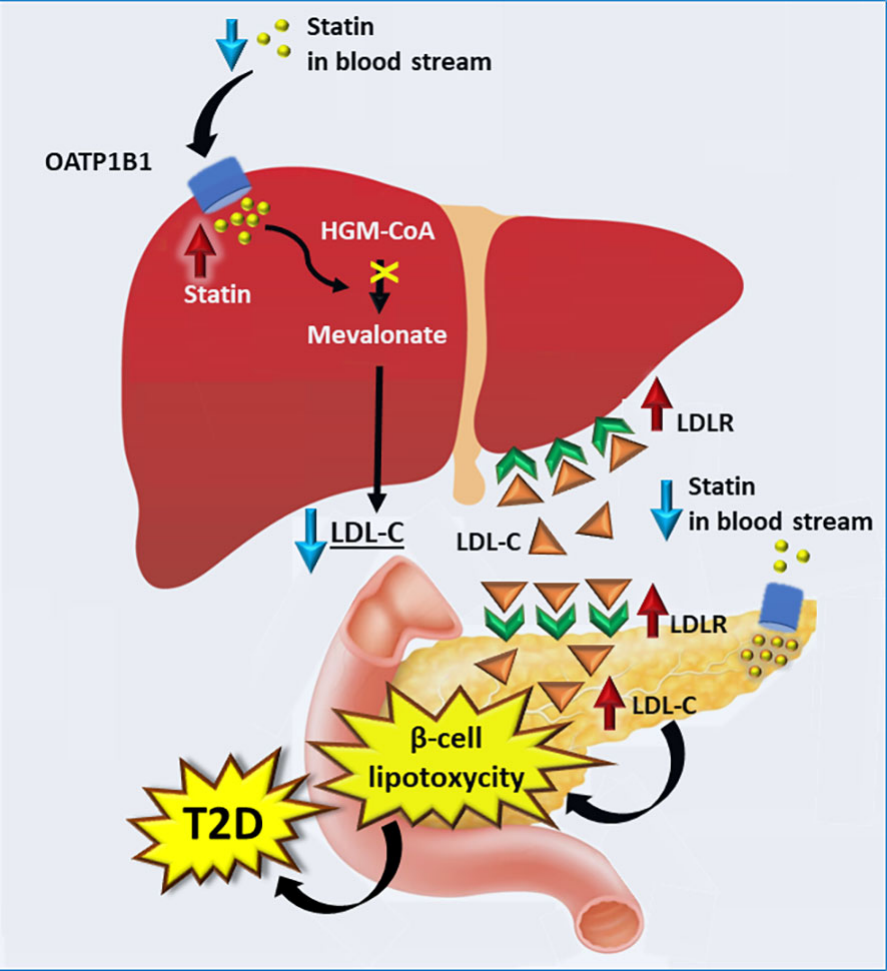
Proposed mechanisms by which statins increase the risk of type 2 diabetes. Statins are transported into the liver by OATP1 resulting in an increase in the LDLR expression and consequently higher absorption of LDL-C into the liver. Expression of LDLR in pancreas is increased upon statin intake, leading to higher concentration of LDL-C. High concentrations of LDL-C in the pancreas results in lipotoxycity in β-cells, decreasing insulin secretion and contributing to the development of T2D. LDLR, low density cholesterol receptor; T2D, type 2 diabetes.

**Figure 2** illustrates possible mechanisms how statins impair insulin secretion. Statins are taken into the liver by transporter protein OATP1B1. In the liver statins inhibit HGM-CoA resulting in downregulation of the mevalonate pathway and increases in *LDLR* expression and LDL-C concentrations. High concentrations of LDL-C are toxic in the pancreatic beta-cells and lead to impaired insulin secretion in the pancreas, and finally hyperglycaemia and T2D. This mechanism is supported by a mouse model lacking LDLR. In this model pancreatic β-cells were protected from accumulation of cholesterol and consequently β-cell function was not impaired (82).

## 8 *In vitro* and *in vivo* studies on mechanisms leading to impaired insulin resistance

Statins increase insulin resistance, but molecular mechanisms are largely unknown. Insulin resistance is found in several tissues, especially in skeletal muscle, liver, and adipose tissue. Insulin resistance itself does not result in the conversion to T2D because impaired insulin secretion is always needed to generate hyperglycaemia. However, long-lasting insulin resistance can contribute to the conversion to T2D if insulin secretion does not compensate insulin resistance.

Skeletal muscle is the main site for insulin-stimulated glucose uptake. Glucose uptake into skeletal muscle is mediated by glucose transporter GLUT4. Grundwald et al. (83) investigated expression of proteins related to GLUT4 mediated glucose uptake in human skeletal muscle tissue from patients on statin treatment. They demonstrated that a short-term statin treatment with statins affected AMPKα and AKT activity in human skeletal-muscle primary myotubes. The conclusion of this study was that AMPKα activation by statins and the blocking of AKT may lead to insulin resistance.

Our metabolomics study showed that genetic inhibition of HMG-CoA reductase was negatively correlated with sphingomyelins and phosphatidylcholines, which increase insulin resistance (15). Sarsenbayeva et al. (84) investigated the association of genetic or pharmacological HMG-CoA reductase inhibition in adipose tissue and concluded that high concentrations of simvastatin decreased adipocyte glucose uptake. They also found a positive association of HMGCR with the insulin signalling pathway, suggesting that reduced activity of HMGCR may contribute to insulin resistance.

Hye Jin Wang et al. (85) found that statin treatment contributed to the development of T2D in mice. Statin treatment (rosuvastatin, atorvastatin, fluvastatin, pravastatin) upregulated the gene expression of key enzymes involved in hepatic gluconeogenesis (*G6PC* and *PCK1*) resulting in increasing glucose production in the liver, and hepatic insulin resistance. Interestingly, these effects were mediated through autophagy induction in the liver.

**Figure 3:**
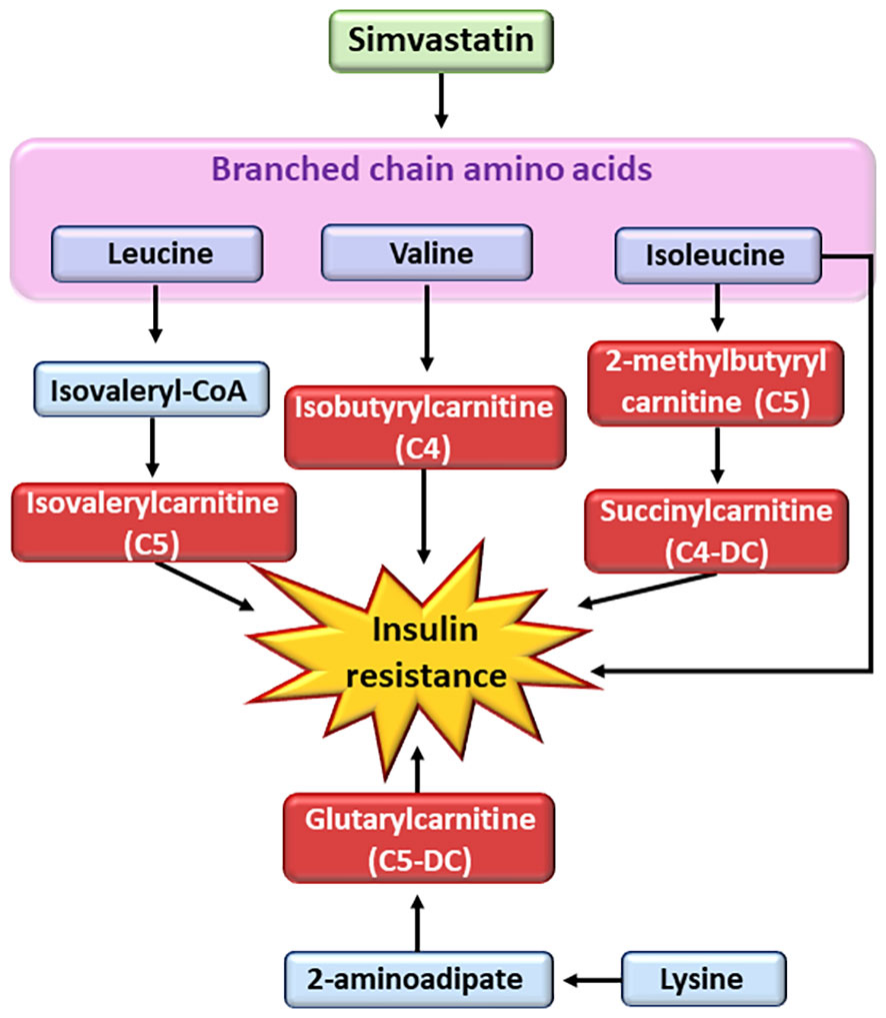
Effects of simvastatin on metabolism of branched chain amino acids. Simvastatin affects the metabolism of branched chain amino acids resulting in the generation of short-chain acyl carnitines (C4 and C5). Short-chain acyl carnitines are associated with insulin resistance. CoA, coenzyme A.

Statins upregulate mitochondrial acylcarnitine carrier gene expression (86). However, detailed metabolite profile of individual acylcarnitines remains unknown. Previous studies have shown that branched-chain amino acids are associated with insulin resistance and the risk of T2D (86–89). We found in the participants of the METSIM study that simvastatin increased concentrations of fife short-chain mitochondrial acylcarnitines, two valine derived metabolites (isobutyrylcarnitine C4, isovalerylcarnitine C5), two isoleucine derived metabolites (2-methylbutyrylcarnitine C5, succinylcarnitine C4-DC), and one lysine derived metabolite (glutarylcarnitine, C4-DC) (15) (**Figure 3**). Additionally, these participants also had increased fasting and 2h glucose concentrations, decreased insulin sensitivity and insulin secretion compared to participants who were not on statin treatment.

In summary, several studies have been published on the association of statins with insulin resistance in skeletal muscle, adipose tissue, and liver but exact mechanisms how insulin resistance is increased are still largely unknown.

## 9 Clinical implications of statin treatment

Several randomised trials have shown that statins lower effectively LDL-C concentrations and the risk of CVD events (coronary deaths, coronary revascularisations, myocardial infarctions, and strokes). It has been estimated that an effective statin treatment for about 5 years in 10 000 patients typically prevents major CVD events in about 1 000 (10%) patients at high risk of heart attacks and strokes (secondary prevention) and 500 (5%) patients at lower risk (primary prevention) (13). There are no significant differences with respect to cardiovascular outcomes between hydrophilic statins (simvastatin, fluvastatin, lovastatin, atorvastin) and hydrophilic statins (rosuvastatin, pravastatin) (90). Adverse effects of statin treatment are myopathy (muscle pain or weakness), incident T2D, and haemorrhagic strokes. It has been evaluated that the treatment of 10 000 patients for 5 years with a standard statin treatment, causes about 5 cases of myopathy, 50–100 new cases of T2D, and 5–10 haemorrhagic strokes (13).

Statins are effective in the lowering not only the risk of CVD, but also microvascular complications. Nielsen et al. (91) demonstrated that microvascular complications decreased during the statin treatment among 213 974 individuals with diabetes from Denmark aged 40 years or older. The follow-up of their study was 215 725 person-years. Compared with individuals without statin treatment, individuals on statin treatment had a 40% lower risk of diabetic retinopathy, 34% lower risk of diabetic neuropathy, and 12% lower risk of gangrene of the foot. However, statin treatment did not lower the risk of diabetic nephropathy.

Several studies available show compelling evidence that statin treatment has more benefit in the prevention of CVD than potential harm related to increased risk of T2D. Diabetes risk is therefore not a reason to withhold statin treatment. Several risk factors for T2D, obesity, blood pressure, total triglycerides, and smoking increase the risk for the conversion to diabetes, and therefore beneficial lifestyle also helps to prevent T2D.

## 10 Concluding remarks

Meta-analyses of clinical statin trials to lower cardiovascular events reported an increase in the risk of diabetes by 9-12% (24, 25, 27) whereas in large population studies the risk was substantially higher. In a meta-analysis of 15 observational studies the risk of incident T2D was 55% (44). In four individual studies the risk of incident T2D was from 36-48% (38, 40, 42, 43), and in the US Veterans study (39) 21-22% for low and median potency statins (pravastatin, fluvastatin), and 34% for high potency statins (simvastatin, atorvastatin, rosuvastatin). Clinical statin treatment trials have significantly underestimated the effects of statins on the risk of T2D. Additionally, the diagnosis of T2D has not performed according to internationally accepted criteria leading to underestimation of incident T2D. In the clinical trials the risk of T2D has been quite similar between different statins but in the population studies statin potency has been a significant factor increasing the risk of T2D.

Recent studies have clarified the significance of the genetic variants as regulators of statin function. A recent study showed for the first time that a variant of the *SLCO1B1* gene encoding OATP1B1-dependent transport of statins in the liver did not have significant association with the risk of T2D (48) whereas variants of the *HMGCR* gene was associated with a 2-6% elevated risk of T2D (50). Interestingly, the pathogenic mutations of the *LDLR* gene increase LDL-C concentration causing familial hypercholesterolemia and are associated with a low risk of T2D, although this association has not be proven to be causal.

Cellular mechanisms how statins increase the conversion to T2D are largely unknown. Impaired insulin secretion is needed to generate hyperglycaemia and the conversion to T2D. The role of insulin resistance can contribute to the conversion to T2D if insulin secretion does not compensate insulin resistance. Statins inhibit HGM-CoA resulting in downregulation of the mevalonate pathway and increases in LDL-C concentrations which are toxic in the pancreatic beta-cells and lead to impaired insulin secretion, and finally to incident T2D. Understanding cellular mechanisms leading to statin-induced incident T2D is the most important challenge in future studies.

## Author contributions

ML and LF wrote the manuscript and performed literature search. This work has not been published or submitted for publication elsewhere. All authors contributed to the article and approved the submitted version.
